# Successful Addition of Topical Tapinarof for Residual Psoriatic Lesions During Apremilast Therapy: A Case Report

**DOI:** 10.7759/cureus.112995

**Published:** 2026-07-20

**Authors:** Nao Kawano, Chisa Nakashima, Atsushi Otsuka

**Affiliations:** 1 Dermatology, Kindai University Hospital, Sakai, JPN

**Keywords:** apremilast, aryl hydrocarbon receptor (ahr), phosphodiesterase 4 inhibitors, psoriasis, tapinarof 1% cream

## Abstract

In some patients with psoriasis, residual skin lesions persist despite systemic treatment with apremilast. However, switching to biological agents may be challenging due to comorbidities, or dose reduction may be required due to side effects, leading to relapses. We report the case of an 86-year-old male with plaque psoriasis and psoriatic arthritis who achieved complete clearance after the addition of topical tapinarof to his apremilast regimen. Although his skin and joint symptoms initially improved with apremilast, mild residual cutaneous lesions recurred after three years (psoriasis area and severity index (PASI) 2.4). Due to a history of gastric cancer, the patient declined biologics. Following the initiation of topical tapinarof, PASI 0 was achieved within 1.5 months. This improvement allowed for a stepwise tapering of both agents; apremilast was eventually reduced to 30 mg every other day (QOD), and tapinarof application was reduced to QOD. The patient has maintained PASI 0 for over one year without recurrence of treatment-related adverse events. The complementary actions of systemic phosphodiesterase 4 inhibition and local aryl hydrocarbon receptor activation, along with tapinarof’s durable remittive effect, may have facilitated this successful long-term remission despite the reduced dosage. However, as this is a single-case observation involving mild residual disease (PASI 2.4), the findings should be interpreted with caution. It remains possible that the topical treatment alone or the low baseline disease burden contributed to the rapid response. Further large-scale studies are needed to confirm the efficacy, safety, and generalizability of this combination strategy.

## Introduction

Psoriasis is a chronic inflammatory skin disease prevalent worldwide, in which interleukin (IL)-17 and IL-23 pathways play a central role in its pathogenesis. It is also associated with several significant comorbidities, including depression, psoriatic arthritis (PsA), and cardiometabolic diseases. Therefore, it is crucial to treat patients at an early stage, identify and prevent comorbidities, promote lifestyle modifications, and provide individualized treatment tailored to each patient [1,2].

Current treatment options for psoriasis include topical therapies, phototherapy, oral treatments such as methotrexate, acitretin, cyclosporine, and the phosphodiesterase 4 inhibitor apremilast, as well as biologic agents [3]. Treatment should be individualized according to disease severity, comorbidities, treatment goals, and patient preferences.

However, managing psoriasis in elderly patients with comorbidities often presents a therapeutic challenge, especially when biologic agents are avoided due to safety concerns. Although apremilast may not match biologic agents in terms of the rapid onset of action or the degree of clinical efficacy, it serves as a valuable therapeutic option for elderly patients with refractory psoriasis who frequently present with contraindications to biologic agents, such as latent tuberculosis infection, viral hepatitis, or a history of malignancy [4]. Furthermore, while topical corticosteroids offer high clinical efficacy, their long-term use can be limited by local adverse effects such as skin atrophy [5].

In contrast, tapinarof is a recently approved non-steroidal topical aryl hydrocarbon receptor (AhR) agonist for the treatment of plaque psoriasis. Clinical trials, including the phase 3 PSOARING studies, have demonstrated the efficacy of tapinarof in patients with plaque psoriasis. In addition, no skin atrophy has been reported to date [6]. Consequently, it may represent an accessible therapeutic option for elderly patients who often exhibit age-related skin fragility. 

Despite the potential benefits of combination therapy, clinical evidence regarding the concomitant use of apremilast and tapinarof remains extremely limited. Establishing the safety and efficacy of such an approach is clinically important, especially for patients who are not candidates for biologic therapy but require additional control for residual lesions.

Here, we report a case of an elderly patient with plaque psoriasis and PsA with a history of early gastric cancer, where the addition of tapinarof not only achieved complete clearance but also allowed for a substantial dose reduction of systemic apremilast. This case suggests the potential utility of this combination therapy in patients for whom biologic therapy is not preferred. However, evidence regarding the combined use of apremilast and tapinarof remains limited, and the findings should be interpreted in light of the limitations inherent to a single-case observation.

## Case presentation

An 86-year-old male with a 40-year history of psoriasis presented with worsening cutaneous lesions. He had been treated with topical corticosteroids for many years. At the initial visit, scattered erythematous lesions with scaling were observed on the trunk and extremities. Treatment with oral etretinate was initiated, resulting in temporary improvement; however, the patient subsequently experienced repeated relapses. Fourteen years after the initiation of etretinate therapy, the patient developed pain in the right Achilles tendon. Musculoskeletal ultrasound revealed bone erosion at the insertion of the right Achilles tendon, along with tendon calcification. In addition, a positive power Doppler signal was detected, leading to a diagnosis of PsA. Following the initiation of apremilast (60 mg/day) and a topical calcipotriol hydrate/betamethasone dipropionate ointment, both skin and joint symptoms initially resolved. The improvement of PsA was determined by clinical assessment, based on the resolution of the patient's Achilles tendon pain. The patient remained on continuous apremilast therapy (60 mg/day). 　　　　

However, three years later, erythematous plaques with scaling recurred on his lower limbs and buttocks without recurrence of joint symptoms (Figures 1a, 1b; psoriasis area and severity index (PASI) 2.4).　

**Figure 1:**
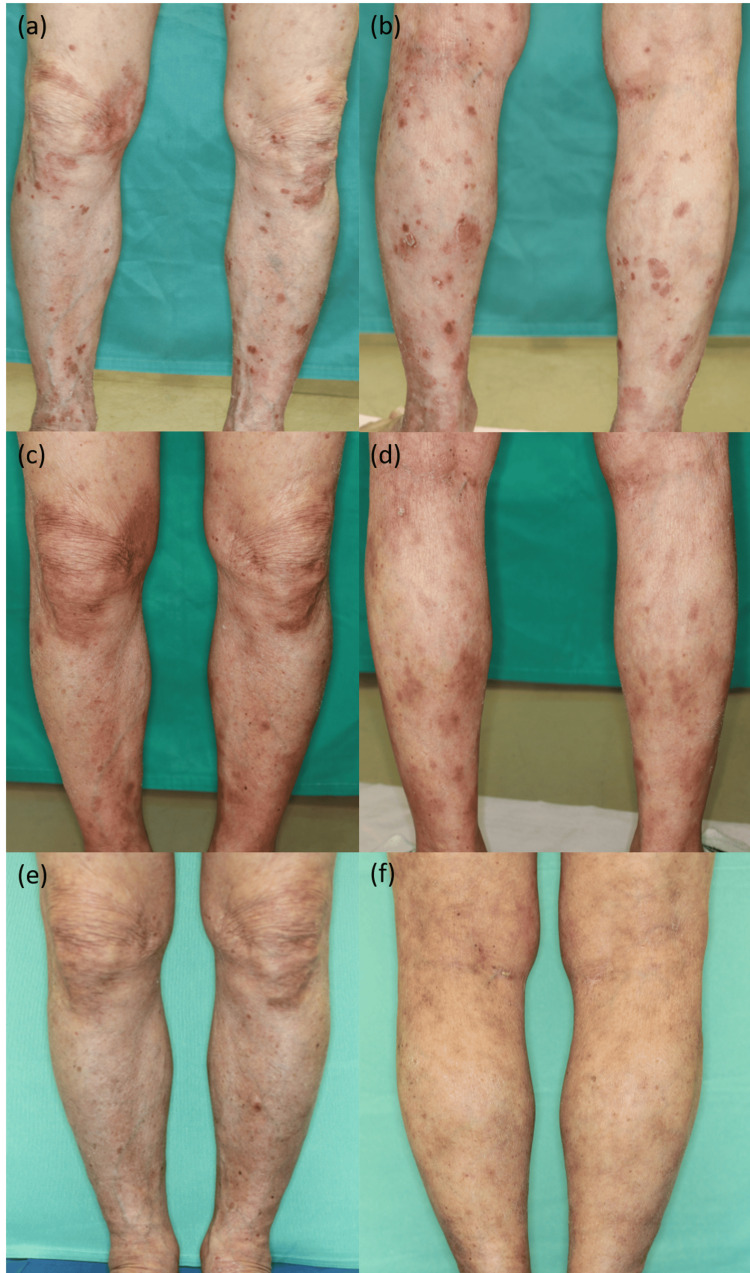
Clinical course before and after the addition of topical tapinarof (a, b) Recurrence of psoriasis before the initiation of topical tapinarof. Multiple erythematous plaques with fine scaling are observed on the lower limbs (psoriasis area and severity index (PASI) score: 2.4). (c, d) Clinical appearance 1.5 months after the addition of topical tapinarof 1% once daily. Complete resolution of the erythema and scaling is noted, achieving a PASI score of 0. (e, f) Sustained remission at the one-year follow-up. Despite tapering systemic apremilast to 30 mg every other day and reducing the frequency of topical tapinarof, the patient has maintained a PASI score of 0.

The patient had been diagnosed with early gastric cancer two years previously and had undergone curative endoscopic submucosal dissection for a 15-mm lesion (pT1a, Stage IA). He remained under regular gastroenterological surveillance with periodic endoscopy, and no evidence of recurrence had been detected. Although biologics were considered, the patient strongly declined them due to concerns regarding the potential impact on his cancer surveillance.

While continuing apremilast, we switched the topical treatment to tapinarof 1% cream once daily because treatment with calcipotriol hydrate/betamethasone dipropionate ointment was insufficient to control the recurrent lesions. Complete clearance (PASI 0) was achieved within 1.5 months (Figures 1c, 1d).

After PASI 0 had been maintained, the apremilast dose was gradually reduced as an individualized treatment decision, taking into account the patient's sustained clinical remission and financial burden. At four months, the apremilast dose was reduced to 30 mg per day and tapinarof application was reduced to every other day (QOD). At nine months, apremilast was further tapered to 30 mg QOD. Remarkably, the patient has maintained PASI 0 for over one year (Figures 1e, 1f) while continuing tapinarof QOD (Figure 2).

**Figure 2:**
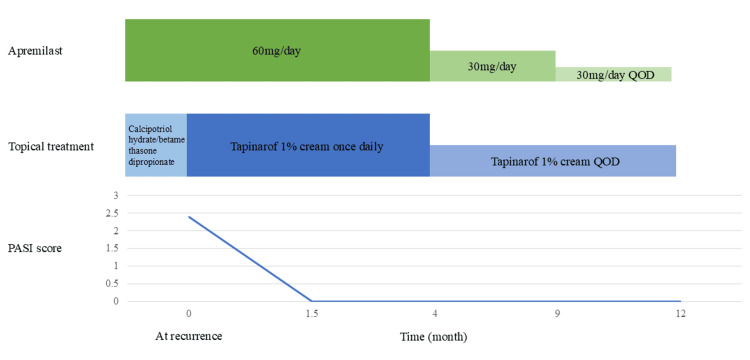
Clinical timeline following the recurrence of psoriasis lesions At the time of relapse (Month 0), the psoriasis area and severity index (PASI) score was 2.4 despite ongoing treatment with systemic apremilast 60 mg/day. Following the initiation of topical tapinarof 1% once daily, complete clearance (PASI 0) was achieved by Month 1.5. At Month 4, apremilast dose was tapered to 30 mg/day and tapinarof application was adjusted to every other day (QOD). At Month 9, apremilast dose was further reduced to 30 mg QOD. Sustained remission (PASI 0) has been maintained for over 12 months without any recurrence of psoriatic arthritis symptoms.

Adherence to topical therapy was assessed by patient interview at each follow-up visit, and no rescue topical corticosteroids or vitamin D/corticosteroid combination therapy was required. The absence of PsA recurrence was determined by clinical assessment; no joint pain, dactylitis, swelling, enthesitis, or functional deterioration was observed during the follow-up period.

Importantly, no treatment-related adverse events, such as gastrointestinal distress, folliculitis, contact dermatitis, irritation, or pruritus, were observed during the treatment with apremilast, tapinarof, or their combination.

## Discussion

Apremilast regulates systemic inflammation by inhibiting phosphodiesterase 4, thereby increasing intracellular cyclic adenosine monophosphate and suppressing pro-inflammatory cytokines such as IL-17 and IL-22 [7]. Additionally, apremilast has been reported to increase the population of IL-10-producing regulatory B cells, an anti-inflammatory subset, while decreasing T helper 1 cells, interferon-gamma-producing natural killer T (NKT) cells, and IL-17-producing NKT cells [8]. In contrast, tapinarof activates the AhR pathway, which suppresses IL-17 production and enhances skin barrier function by upregulating filaggrin and loricrin expression [9]. Furthermore, tapinarof partially induces genes associated with the nuclear factor erythroid 2-related factor 2 pathway, which promotes antioxidant responses in the skin by scavenging reactive oxygen species [10]. Given these distinct pharmacological pathways, the combination of systemic apremilast and local tapinarof may provide complementary therapeutic effects, targeting both systemic immune dysregulation and the local skin microenvironment.

Furthermore, it has been reported that AhR activation induces regulatory T cells and downregulates the activity of resident memory T cells, which are key drivers of local relapse [11,12]. Clinical trials have demonstrated a durable remittive effect of tapinarof; in PSOARING 3, the mean duration of the off-therapy remittive effect among patients achieving physician global assessment (PGA) 0 was 130.1 days [6]. Because our patient continued treatment with both apremilast and tapinarof, the sustained remission observed in this case cannot be interpreted as equivalent to the off-therapy remittive effect reported in PSOARING 3. Nevertheless, this durable remittive effect may have contributed to improved control of the cutaneous lesions, thereby enabling successful tapering of systemic apremilast in our patient.

However, several limitations must be acknowledged. As this is a single-case observation, definitive conclusions regarding causality or universal synergistic effects cannot be established. The baseline disease activity at the time of tapinarof initiation was relatively mild (PASI 2.4). Consequently, we cannot exclude the possibility that the residual lesions responded to tapinarof monotherapy alone, regardless of the ongoing apremilast treatment, or that the low initial disease burden facilitated the rapid achievement of PASI 0. Furthermore, spontaneous fluctuations in psoriasis severity or a delayed, cumulative effect of long-term apremilast therapy might have influenced the clinical course.

Despite these limitations, the use of tapinarof as an adjunctive therapy is gaining attention. A recent case report described complete clearance of residual psoriatic lesions following the addition of topical tapinarof to ongoing tildrakizumab therapy [13]. This observation suggests that tapinarof may be a useful adjunctive treatment for residual lesions; however, our observation of successful systemic drug tapering adds a novel perspective to this strategy.

The efficacy observed in our patient is consistent with the PSOARING 1 and 2 trials, where 14% of patients achieved PGA 0/1 by Week 4 [14], and the long-term extension study PSOARING 3, where 40.9% achieved PGA 0 at least once [6]. Our patient’s sustained PASI 0 for over one year while on reduced dosages of both agents suggests that this combination may represent a viable long-term maintenance strategy.

Nevertheless, larger controlled studies are required to validate the safety and efficacy of this approach and to determine its generalizability to patients with more severe residual disease before recommending it as a standard dose-sparing strategy.

## Conclusions

In the present case, the addition of topical tapinarof successfully cleared residual psoriasis lesions that had persisted under apremilast-based therapy. Furthermore, this combination enabled a stepwise dose reduction of apremilast while maintaining long-term remission. However, this was a single-case observation involving mild residual disease (PASI 2.4), and the possibility of a response to topical therapy alone or other contributing factors must be considered. The combination of apremilast and tapinarof may represent a potential therapeutic option deserving further investigation in larger, controlled studies to evaluate its efficacy, safety, and broad clinical applicability.
